# Gastric volvulus and wandering spleen in Pitt–Hopkins syndrome: first paediatric case report

**DOI:** 10.3389/fped.2025.1705547

**Published:** 2025-11-25

**Authors:** Gabriele Vasta, R. Angotti, G. Contini, M. L. Perrotta, F. Molinaro, L. Tarallo, C. Plessi, V. Briganti

**Affiliations:** 1 Paediatric Surgery, San Camillo-Forlanini Hospital, Rome, Italy; 2 Division of Paediatric Surgery, Department of Medical, Surgical and Neurological Sciences, University of Siena, Siena, Italy

**Keywords:** gastric volvulus, wandering spleen, Pitt–Hopkins syndrome, laparoscopic gastropexy, paediatric surgery case report

## Abstract

**Background:**

Acute gastric volvulus is a rare condition in children, and delayed diagnosis may lead to gastric ischemia, perforation, or even death. It is sometimes associated with a wandering spleen, a condition in which the spleen migrates from its normal anatomical position due to the absence of fixation ligaments. We report the first known case of a patient with Pitt–Hopkins syndrome (PTHS) presenting with simultaneous acute gastric volvulus and a wandering spleen.

**Case report:**

A 6-year-old male with PTHS was urgently referred for acute abdominal pain and a 24 h history of non-bilious and non-bloody emesis. The x-ray showed a massive gastric dilatation, and an upper gastrointestinal series (UGI) revealed a gastric outlet obstruction. An emergency laparoscopy revealed a gastric mesoaxial volvulus with a hypotonic wall with no sign of ischemia or perforation associated with a wandering spleen. Gastropexy was performed by anchoring the gastric greater curvature to the anterior abdominal wall, covering the spleen in a good position in the left upper abdomen, completely covered by the gastric fundus. The patient made an uneventful recovery and was completely asymptomatic.

**Conclusion:**

To our knowledge, this is the first reported case of simultaneous gastric volvulus and wandering spleen in a patient with PTHS. Laparoscopic gastropexy is a straightforward and effective procedure that combines the advantages of previously described surgical techniques.

## Introduction

Gastric volvulus denotes the rotation of either the entirety or a segment of the stomach by at least 180° around an axis, leading to an obstruction within the foregut. This obstruction could manifest as acute, recurrent, intermittent, or chronic. There are three known types of gastric volvulus: *organoaxial volvulus*, *mesenteroaxial volvulus*, and *combined volvulus* (1). Wandering spleen is also a rare condition characterized by a migration of the spleen from its usual anatomical position, commonly to the lower abdomen or pelvis. Both defects are a result of the incomplete formation of the intraperitoneal suspensory ligaments and can sometimes be associated with surgical complications (2, 3). Their association is rare, but known in the literature, with several cases reported in the adult population (4–13) and also in the paediatric population (8, 14–20).

Pitt–Hopkins syndrome (PTHS) is a neurodevelopmental disorder with physical, cognitive, and behavioral characteristics, caused by deletions of or variants in the TCF4 gene located at 18q21.2, which encodes transcription factor 4 (21). Reliable prevalence figures have not been published, but based on the number of known affected individuals in the United Kingdom and the Netherlands, prevalence is estimated as one in 225,000–300,000 (22).

Patients with Pitt–Hopkins syndrome present with feeding difficulties, often constipation (80%–70%), and gastroesophageal reflux. Episodes of hyperventilation may cause aerophagia, resulting in excessive eructation and abdominal distension, which can be associated with discomfort or pain (23, 24).

In this paper, we report the first case report, to our knowledge, of simultaneous gastric volvulus and wandering spleen in a patient with Pitt–Hopkins syndrome.

## Case description

A 6-year-old male with Pitt–Hopkins syndrome was urgently referred to our clinic for acute abdominal pain associated with a 24 h history of non-bilious and non-bloody emesis. The symptoms were also associated with lethargy and abdominal distension. He had no history of fever, diarrhea, toxic ingestion, or trauma.

**Figure 1:**
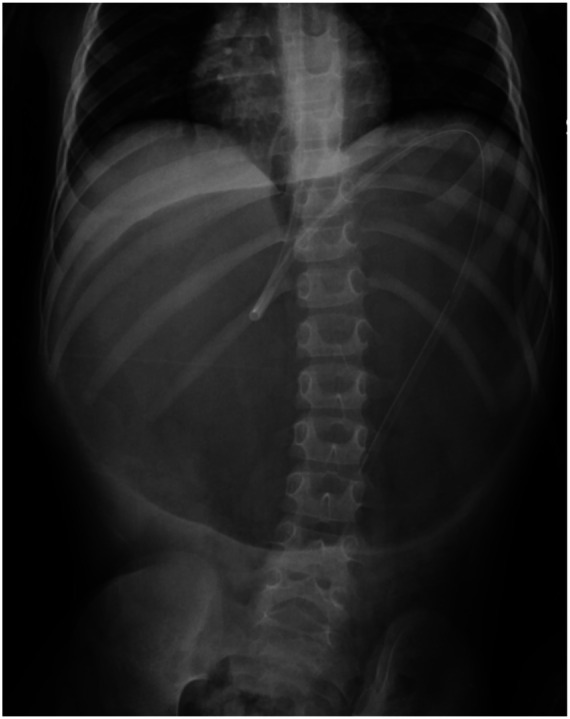
Plan abdominal x-ray showed massive gastric dilatation, NG tube in place.

On physical examination, he showed signs of dehydration, including dry mucous membranes and reduced skin turgor, as well as a firm, non-tender epigastric mass on abdominal palpation. A plain abdominal x-ray showed a massive gastric dilatation (Figure 1).

After intravenous resuscitation, a nasogastric tube was inserted, initially draining approximately 100–200 cc of gastric material, after which a second plain abdominal x-ray was performed and confirmed persistent gastric distension.

Since the stomach remained markedly dilated, a further aspiration was carried out, draining approximately an additional 1,000 cc of air and gastric content. At this stage, given the strong suspicion of gastric volvulus already raised by the radiographic findings and the significant decompression achieved, a CT scan was not performed. The patient remained hemodynamically stable, and as the radiologist felt confident in proceeding, an upper gastrointestinal (UGI) contrast study was performed to further clarify the diagnosis.

**Figure 2:**
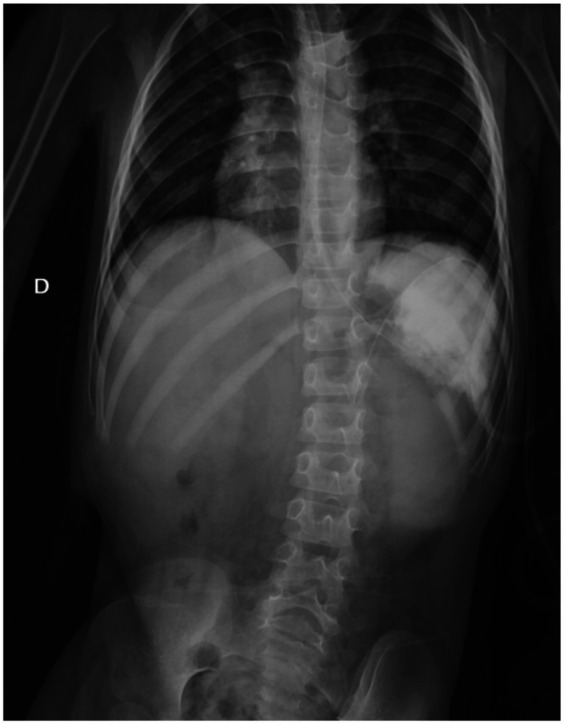
Plan abdominal x-ray after aspiration and UGI contrast showed partial rotation of the greater curvature toward the diaphragmatic side.

A barium study (Figure 2) demonstrated a partial rotation of the greater curvature toward the diaphragmatic side, raising suspicion of gastric volvulus, also supported by delayed gastric emptying. After intravenous resuscitation, the nasogastric tube was inserted, draining approximately 1,200 cc of gastric content with no evidence of bilious fluid. A repeat plain abdominal x-ray was performed, confirming the persistence of the volvulus.

**Figure 3:**
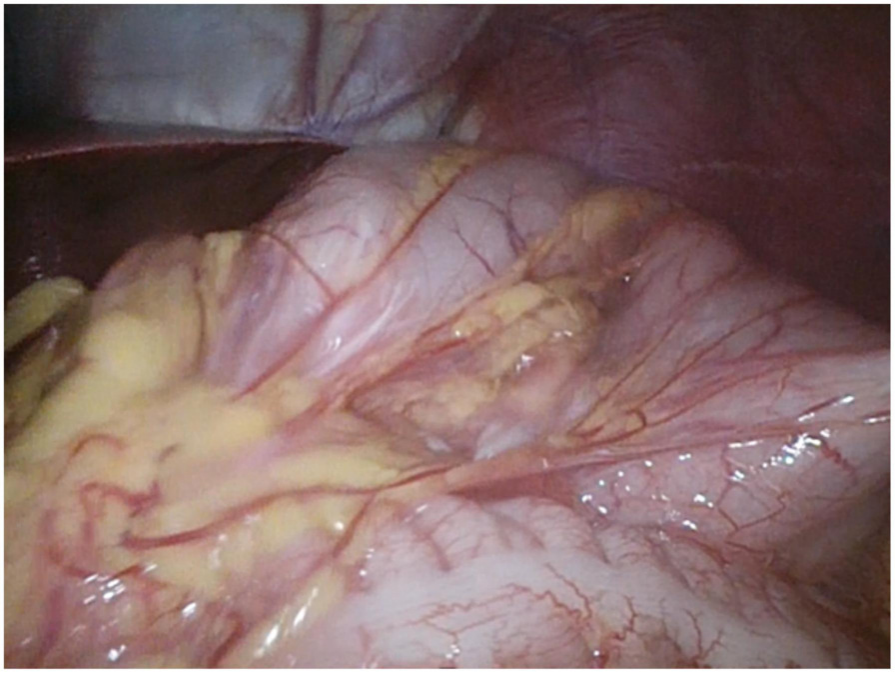
Laparoscopic identification of gastric volvulus.

**Figure 4:**
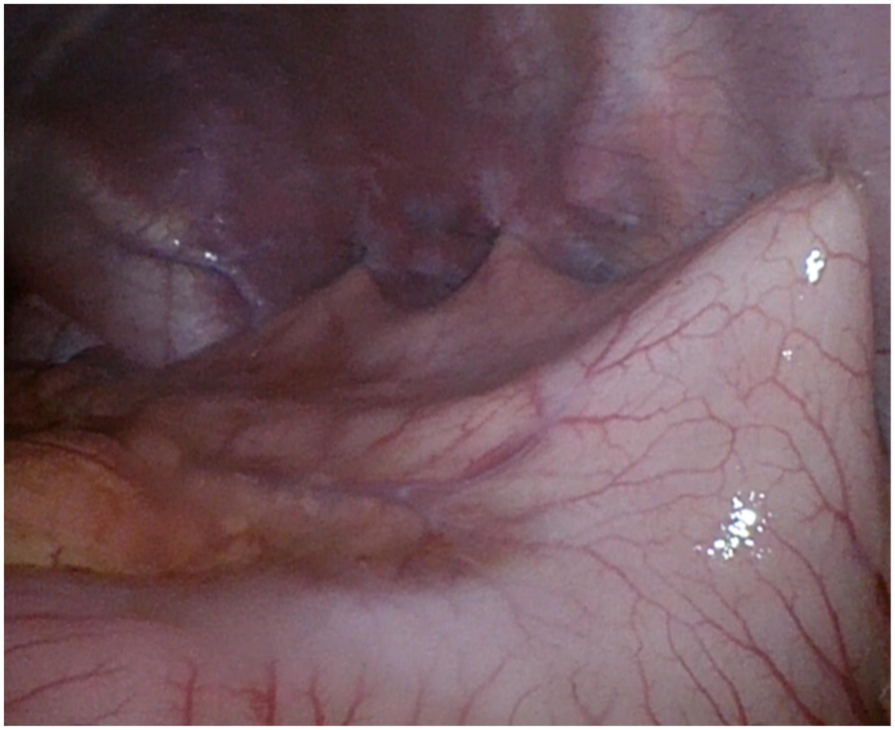
Anterior laparoscopic gastropexy.

Due to the persistence of episodes of vomiting and the severe clinical condition, an urgent laparoscopic exploration was performed. A 10 mm trocar was inserted trans-umbilically, using *the Hasson approach*. A laparoscope (30°) was inserted through the umbilical port. Laparoscopic exploration revealed a gastric mesoaxial volvulus with a hypotonic wall with no sign of ischemia or perforation, associated with a wandering spleen, augmented in volume without normal supporting ligaments. The volvulus was promptly reduced (Figure 3), and the stomach began to regain its normal color almost immediately. The abdomen was copiously irrigated, and all other abdominal viscera were inspected and appeared normal. The diaphragm was examined on both sides and found to be normal. Faced with splenic viability, we decided to perform an isolated anterior gastropexy without splenectomy. Three additional working ports were inserted: left side and right side of the umbilicus and epigastrium. The spleen was then moved freely from its abnormal location (anterior abdominal wall) to its normal one (subdiaphragmatic). Gastropexy was performed by fixing the gastric greater curvature to the anterior abdominal wall, covering the spleen in a good position in the left upper abdomen, completely covered by the gastric fundus (Figure 4).

**Figure 5:**
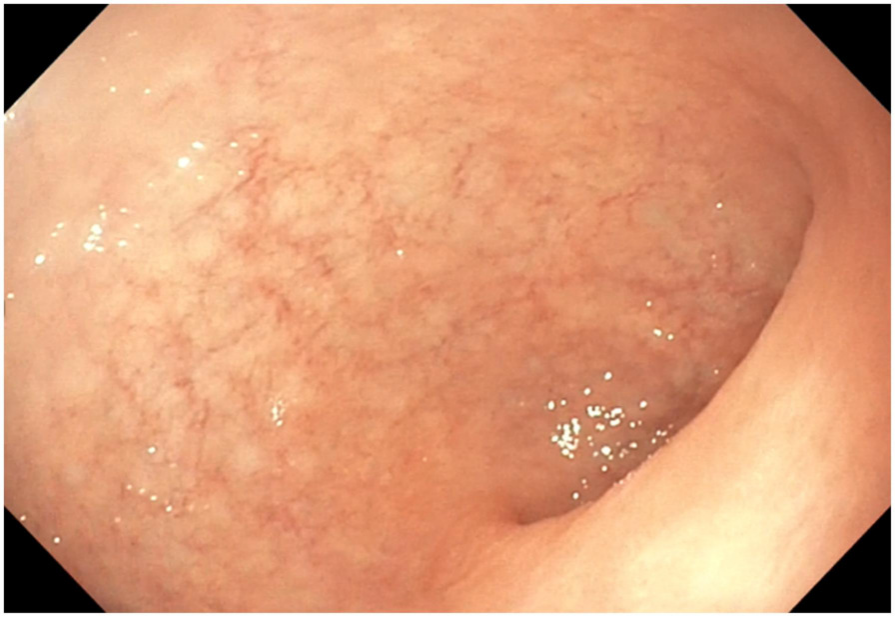
Oesophago-gastro-duodenoscopy (OGD) after surgery showed no tissue damage to the stomach.

At the end of the procedure, we performed a gastroscopy with a flexible paediatric gastroscope to check the gastric mucosa. No tissue damage was found in the esophagus or inside the stomach (Figure 5). The duodenum showed no signs of tissue distress. Additionally, the orientation of the stomach was correct. This intraoperative gastroscopic evaluation also allowed direct assessment of gastric vitality, confirming the absence of ischemic or necrotic changes.

Postoperatively, the patient was transferred to the paediatric critical care unit for observation. Five days after surgery, the patient was discharged home without further complications. At the last follow-up 12 months after surgery, the patient was in good clinical condition, without further history of vomiting or any other symptoms.

At long-term follow-up, no impairment of gastric peristalsis was observed, and the patient maintained a normal feeding pattern, suggesting that the anterior gastropexy did not adversely affect gastric motility.

## Discussion

Gastric volvulus is infrequent, with the incidence peaking after the fifth decade and adults comprising 80%–90% of the cases. It can be classified based on its etiology as primary or secondary. Primary (idiopathic) gastric volvulus is characterized by abnormalities of the gastric ligaments, such as agenesis, elongation, or disruption. Secondary gastric volvulus is observed in approximately two-thirds of patients with gastric volvulus and is attributed to various anatomical abnormalities, including paraesophageal hernia, diaphragmatic hernia or eventration, and phrenic nerve paralysis.

Additionally, anatomic abnormalities affecting other organs, especially the stomach or spleen, can also contribute to secondary gastric volvulus (25).

Wandering spleen is a rare condition characterized by a migration of the spleen from its usual anatomical position, commonly moving to the lower abdomen or pelvis. Its exact incidence is unknown and is likely underestimated. The peak incidence is reported between the ages of 20 and 40 but can also occur in the paediatric population. Similar to gastric volvulus, this condition is caused by defects in the abdominal ligaments that normally support the spleen (26, 27). Additionally, it has been reported to be associated with congenital defects (such as congenital diaphragmatic hernia or omphalocele) (28).

Rarely, an association between gastric volvulus and wandering spleen has been reported. Both conditions are associated with a failure of fusion of the mesogastrium with the posterior body wall, leading to incomplete formation of splenic supporting ligaments and facilitating gastric rotation (21).

The PTHS is characterized mainly by intellectual impairment, specific facial characteristics, and marked autonomic nervous system dysfunction, mainly involving respiration and intestinal mobility. Gastrointestinal manifestations are frequent and include constipation and gastroesophageal reflux disease. Moreover, hyper breathing due to disrupted regulation of respiration may lead to air swallowing with subsequent excessive burping and pain due to abdominal swelling. Conversely, structural visceral malformations are rarely observed in patients with PTHS (22).

We found no reported cases in the literature of gastric volvulus, with or without associated wandering spleen, in patients with Pitt–Hopkins syndrome. Nevertheless, we identified two cases of gastric volvulus associated with overeating and gastric distension: a 12-year-old girl (29) and a 28-year-old man (30).

Although this is the first reported case in the literature, we can speculate that gastric distension due to air ingestion typical of PTHS may have contributed to the onset of gastric volvulus in a patient with a predisposing anatomical configuration, notably of the abdominal ligaments. As shown in our case, gastric volvulus needs of a prompt diagnosis and treatment to reduce the mortality rate. When the stomach rotates more than 180°, indeed, it leads to complete obstruction of the gastric outlet, potentially resulting in ischemia, strangulation, necrosis, perforation, and abdominal sepsis. Cole and Dickinson (31) documented a mortality rate of 65% in case reports involving children with acute gastric volvulus prior to 1950.

Timely diagnosis is facilitated by recognizing common presentation patterns, such as the sudden onset of persistent, non-bilious vomiting, often accompanied by epigastric discomfort and bloating. Respiratory distress, cyanosis, or hematemesis may occur, albeit less frequently. Chest x-rays may reveal a prominently distended stomach situated at or above the diaphragm level, oriented either horizontally (organoaxial) or vertically (mesenteroaxial). In some cases, even more in patients with PTHS, who often present with gastric distension due to air ingestion in paraphysiologic conditions, further confirmation through advanced radiologic examinations may be necessary. In our case report, the diagnosis was supported by an UGI contrast study. Although a CT scan can also be useful, especially to assess associated anomalies, it is less commonly employed in paediatric patients. In emergency conditions such as gastric volvulus, however, prompt surgical exploration remains mandatory (32).

If gastric volvulus is confirmed and conservative treatment with a nasogastric tube fails, a surgical intervention is needed. Although it can be performed using either open or laparoscopic techniques. The last one has the undeniable advantage of allowing exploration of the abdominal cavity for associated malformations.

The choice of the surgical procedure, however, may vary based on associated pathological findings. In fact, although classically, the treatment of wandering spleen involved splenectomy, conservative treatment with splenopexy is now preferred. In 2010, Fiquet-Francois et al. (33) hypothesized that splenopexy might be associated with secondary splenic ischemia, whereas gastropexy alone might have the advantage of not manipulating the spleen.

However, a standardized surgical approach for gastric volvulus associated with a wandering spleen has not yet been established (34).

In our case report, given the spleen's good condition at the time of inspection, we decided to preserve it by repositioning it to its anatomical location in the left hypochondrium. We secured it in place with an anterior gastropexy, which also served to prevent recurrence of the gastric volvulus.

## Conclusion

Gastric volvulus associated with wandering spleen is a rarely described condition in the paediatric age group, being potentially life-threatening if not immediately managed. The classical Borchardt triad is observed in approximately 50% of cases. Plain and contrast radiographs are helpful in the preoperative diagnosis. Laparoscopic exploration in this case has a dual relevance: diagnosis and therapy. Laparoscopic gastropexy for gastric volvulus associated with a wandering spleen is an easy procedure and combines the advantages of all surgical techniques adopted in previous eras. Clinicians caring for patients with Pitt–Hopkins syndrome, given their tendency toward aerophagia, over-breathing, and gastric distension, should consider the possibility of a gastric volvulus in the presence of compatible symptomatology. To our knowledge, this is the first case of gastric volvulus with wandering spleen in a patient affected by Pitt–Hopkins syndrome.
